# Supplementary material for: A whole-animal phenotypic drug screen identifies suppressors of atherogenic lipoproteins
Source: eLife. 2026 Aug 27;14:RP105314. doi: 10.7554/eLife.105314 (PMC13521566; doi:10.7554/eLife.105314)
Supplement: Supplementary file 2 [file elife-105314-code1.zip › JHDL_primary_result/Peanut oil/Peanut oil_JHDL_30_019_019_2019_12_13_11_22.pdf]

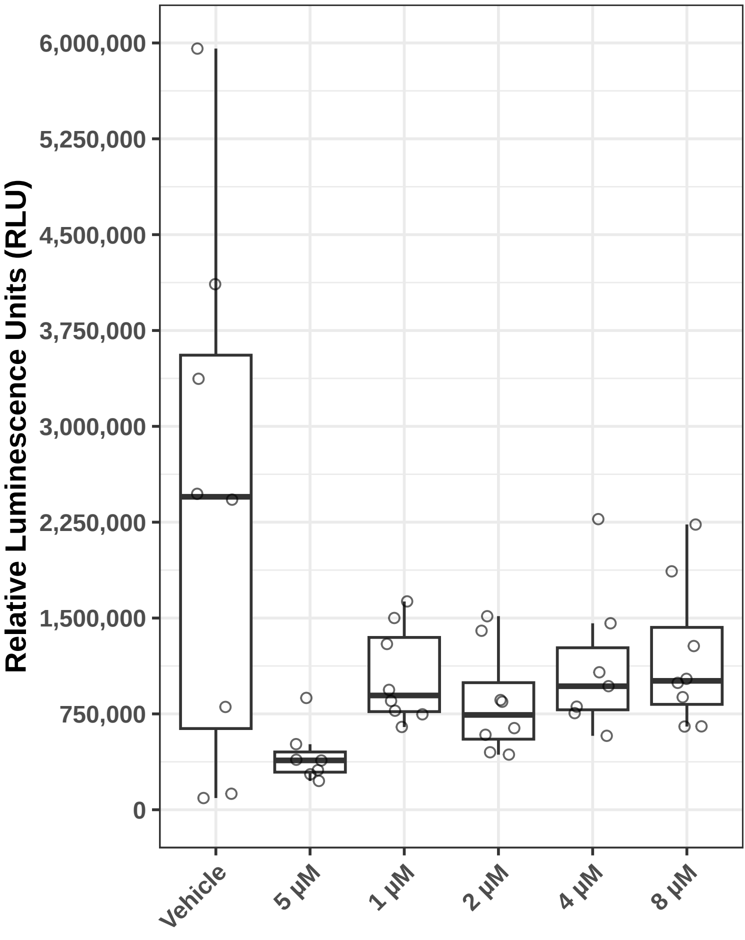
Relative Luminescence Units (RLU)
6,000,000
5,250,000
4,500,000
3,750,000
3,000,000
2,250,000
1,500,000
750,000
0
Vehicle
5 µM
1 µM
2 µM
4 µM
8 µM
